# Telesimulation as a modality for neonatal resuscitation training

**DOI:** 10.1080/10872981.2021.1892017

**Published:** 2021-02-18

**Authors:** Lukas P. Mileder, Michael Bereiter, Thomas Wegscheider

**Affiliations:** a Division of Neonatology, Department of Pediatrics and Adolescent Medicine, Medical University of Graz, Graz, Austria; b Clinical Skills Center, Medical University of Graz, Graz, Austria; c Division of Special Anesthesiology, Pain and Intensive Care Medicine, Department of Anesthesiology and Intensive Care Medicine, Medical University of Graz, Graz, Austria

**Keywords:** Neonate, resuscitation, education, simulation training, telesimulation

## Abstract

**Introduction**: Telesimulation may allow simulationists to continue with essential simulation-based training programs during the COVID-19 pandemic. Hence, we investigated the feasibility of telesimulation for neonatal resuscitation training, assessed participants’ attitudes towards telesimulation as well as its effect on neonatal resuscitation knowledge, and compared results between medical students and neonatal nurses.

**Methods**: For this prospective observational pilot study, medical students and neonatal nursing staff were recruited on a voluntary basis. Pre- and post-training knowledge was assessed using a 20-question questionnaire. Following the educational intervention, participants further answered a six-item questionnaire on their perception of telesimulation.

For the telesimulation session, participants received a simulation package including a low-fidelity mannequin and medical equipment. The one-hour telesimulation session was delivered by an experienced instructor and broadcasted via Cisco Webex for groups of 2–3 participants, covering all elements of the neonatal resuscitation algorithm and including deliberate technical skills practice.

**Results**: Nine medical students and nine neonatal nurses participated in a total of seven telesimulation sessions. In general, participants enjoyed the telesimulation session, acknowledged a positive learning effect and found telesimulation suitable for neonatal resuscitation training, but were critical of potential technical issues, training logistics, and the quality of supervision and feedback. Neonatal resuscitation knowledge scores increased significantly after the educational intervention both for medical students and nurses.

**Conclusions**: Telesimulation is feasible for neonatal resuscitation training and associated with significant improvements in knowledge of current resuscitation guidelines, without differences between medical students and neonatal nurses.

## Introduction

Medical education and training have changed dramatically during the COVID-19 pandemic, urging simulationists to identify novel delivery modes to continue with simulation-based training (SBT) programs. Telesimulation, as an aspect of distance education, offers remote training opportunities, and can be defined as *‘ … process by which telecommunication and simulation resources are utilized to provide education, training, and/or assessment to learners at an off-site location’*[1]. It has been successfully applied to a variety of target groups, contents, and settings[1], yet only few studies have evaluated its effectiveness prospectively. Jain et al [2]. randomized nurses to either receive neonatal resuscitation instruction in the classroom or via tele-instruction – post-training knowledge and skills increased significantly in both groups, yet adjustment for baseline knowledge revealed significantly better performance in both domains in the classroom group. To add to the existing evidence, we investigated (i) the feasibility of telesimulation for neonatal resuscitation training based on participants’ evaluations and (ii) its effect on participants’ neonatal resuscitation knowledge as an objective measure of outcome.

## Materials and methods

We performed a prospective observational pilot study (July 2020 – February 2021). Participants were recruited among student peer-teachers at the Clinical Skills Center, Medical University of Graz, and neonatal nursing staff at the Division of Neonatology Graz, Austria. Participation was voluntary without financial compensation.

In order to evaluate baseline knowledge of neonatal resuscitation guidelines[3], participants answered a 20-question single-choice paper-and-pencil questionnaire [4] together with demographic questions.

For individual home training, participants received a bag containing a low-fidelity mannequin (Newborn Anne™ or Baby Anne™, both Laerdal Medical, Norway, depending on availability), towel, cotton cap, stethoscope, suction catheter, and one neonatal self-inflating ventilation bag with appropriately sized face mask (Laerdal Medical, Norway), and were sent a web link to participate in the telesimulation activity via a personal computer with web camera or smartphone. The one-hour telesimulation session was broadcasted via Cisco Webex from the neonatal resuscitation suite at the Clinical Skills Center. It was delivered by an experienced instructor (LPM) as an algorithm training with open discussion for groups of 2–3 participants, covered all elements of the neonatal resuscitation algorithm[3], and contained deliberate practice of drying, tactile stimulation and opening the airway, bag-valve-mask ventilation, and chest compressions. Participants demonstrated skills consecutively and received individual feedback on task performance.

Following the educational intervention, participants answered a) the same questionnaire [4] (post-test) with answers in random order and b) a six-item questionnaire on their perception of telesimulation using five-point Likert-like scales (five being the highest rating), dichotomous and multiple-choice questions with free-text option, both delivered electronically (www.surveymonkey.com; SurveyMonkey Europe UC, Dublin, Ireland).

### Statistical analysis

We used SPSS Statistics 26 (IBM Corporation, Armonk, USA) to analyse data. Frequencies are displayed using absolute and relative numbers. Continuous variables are given as median (interquartile ranges [IQR]). We used Wilcoxon-rank-sum test to compare pre- and post-training knowledge scores. For comparisons between groups, either Student’s t-test for independent samples or Mann-Whitney-U-test were employed. A p-value of <0.05 was considered statistically significant.

## Results

Nine medical students (median age: 27 [IQR 23.5–28] years; median year of studies: 3 [IQR 2.5–4.5]; m:f = 5:4) and nine members of the neonatal nursing staff (median age: 31 [IQR 24.5–35.5] years; median year of professional work: 6 [IQR 3–12]; m:f = 1:8), five of whom (55.6%) had previous experience with cardiopulmonary resuscitation after birth, participated. A total of seven telesimulation sessions were held successfully, without any relevant issues related to Internet connectivity or audio-video quality.

Participants’ perception of telesimulation is summarized in Table 1. In general, they enjoyed the telesimulation session a lot (median 5, IQR 5–5), admitted that they had gained knowledge (median 4, IQR 4–4.25), found this educational methodology suitable for neonatal resuscitation training (median 4, IQR 4–5), and acknowledged the practical training opportunities (18/18, 100%). The only difference in participants’ perception was that medical students rated their knowledge gain favorably (median 4 [IQR 4–5] vs. median 4 [IQR 3–4], p = 0.010).

**Table 1.** Training participants’ (n = 18) perception of telesimulation (five being the highest rating)

**Statement**	**Rating (five-point Likert-like scale)** **median (IQR)**
I have enjoyed the telesimulation session.	5 (5–5)
I have gained knowledge in the telesimulation session.	4 (4–4.25)
Telesimulation is suitable for neonatal resuscitation training.	4 (4–5)
**Statement**	Yes/No (numbers and percentages)
I had the opportunity to train technical skills in the telesimulation session.	Yes: 18/18 (100%)
Telesimulation offers the same training opportunities as traditional face-to-face instruction.	No: 10/18 (55.6%)
Perceived disadvantages of telesimulation in comparison to traditional face-to-face instruction: Technical difficulties (e.g., network connectivity or sound quality) Logistics related to training package pick-up and return Limited practical training opportunities Others (free text): Limited quality of supervision and feedback due to the physical distance between instructor and trainees	Yes: 14/18 (77.8%) Yes: 7/18 (38.9%) Yes: 4/18 (22.2%) Yes: 4/18 (22.2%)

**Figure 1.**
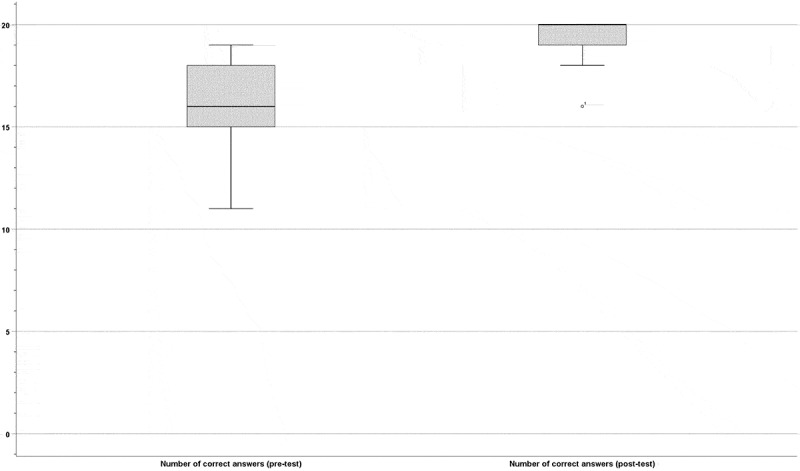
Participants’ knowledge scores (i.e., numbers of correct answers in the 20-question questionnaire) before (left bar) and after (right bar) the educational intervention (p = 0.001)

For the whole cohort of 18 participants, neonatal resuscitation knowledge scores increased significantly from a median of 16/20 (IQR 14.5–18) before to 20/20 (IQR 18.75-20) correct answers after the educational intervention (p = 0.001; Figure 1). There were no differences in pre- and post-test knowledge scores between medical students and neonatal nurses (p = 0.427 and p = 0.418, respectively). In the sub-group of medical students, the number of correct answers increased from a median of 16/20 (IQR 14–17) to 20/20 (IQR 18–20; p = 0.011). Prior to the telesimulation session, nursing personnel had a median of 17/20 (IQR 14–18) correct answers on the questionnaire, compared to a median of 20/20 (IQR 19–20) afterwards (p = 0.018).

## Discussion

By rendering instructor expertise virtually available, telesimulation offers standardized training in remote areas and during times of social distancing and infectiological concerns. Due to local COVID-19 associated training restrictions, we have designed and implemented a telesimulation activity for neonatal resuscitation training. We have utilized this concept several times successfully and can report, based on these preliminary results, that telesimulation for neonatal resuscitation training is feasible from a technical standpoint. This supports findings from other studies, examining the feasibility of telesimulation for robotic [5] and laparoscopic surgery[6], ultrasound-guided regional [7] and pediatric anesthesia[8], emergency medicine[9], pediatric resuscitation[10], intraosseous access[11], and mass casualty triage[12].

Telesimulation was well perceived and participants acknowledged a positive learning effect. However, although we used the concept of deliberate practice, the majority of participants still preferred traditional face-to-face instruction. This finding may be attributed to the brief 60-minute telesimulation session and could potentially be optimized by extending the training time for more dedicated skills practice. Furthermore, 22.2% of participants rated the delivery of instructor feedback critically – although previous investigations have shown that participants perceive remotely facilitated SBT less positively than local facilitation[13], this finding still warrants further study, as feedback is essential for the success of any SBT[14]. Reducing the number of participants and optimizing the audio/video quality, e.g., by using headphones and high-definition cameras, could help conquering this limitation. Yet, despite participants’ critical evaluation of the feedback quality, performance assessments generally do not differ between local and remote simulation facilitators [7,11,15].

We found significantly increased short-term neonatal resuscitation knowledge in the post-training survey. This increase in the cognitive domain supports results from previous studies [11] and adds to the findings by Jain et al. [2].

Costs are a relevant obstacle for implementation of SBT programs[16]. As we utilized existing teleconference software and IT hardware as well as low-fidelity training equipment that was already available at our Clinical Skills Center, the only relevant costs associated with the presented telesimulation curriculum were the invested working hours for participants and the single instructor.

### Limitations

As this was a pilot, hypothesis generating study, we acknowledge our small sample size. At the beginning of our pilot study we had to use Baby Anne (infant) mannequins, as these were the only ones available at this time; for the latest telesimulation sessions we utilized Newborn Anne mannequins. Yet, results using either mannequin were similar in regard to knowledge gain and participants’ perception. Given the lack of a control group, we can only speculate whether telesimulation yields comparable improvements in the cognitive domain in comparison to face-to-face SBT or more traditional educational approaches. Further, we did not examine trainees’ technical skills following the telesimulation session, but evidence suggests that telesimulation is associated with improvements in procedural skills performance [7,10,17].

## Conclusion

In conclusion, telesimulation is feasible for neonatal resuscitation training and associated with significant improvements in knowledge of current resuscitation guidelines both in medical students and neonatal nurses. While we will use participants’ evaluations of our pilot curriculum to further refine it, we are convinced that the presented telesimulation program may be adopted by other educational institutions during times of social distancing both for graduate education and interprofessional postgraduate refresher training.

## Data Availability

Research data is confidential. Deidentified participant data will be made available to researchers only upon reasonable request to the study authors.
